# Evaluation of the correlation between effort-reward imbalance and sleep quality among community health workers

**DOI:** 10.1186/s12913-021-06526-w

**Published:** 2021-05-22

**Authors:** Xuexue Deng, Ronghua Fang, Yaoting Cai

**Affiliations:** Department of International Medical Center, West China Hospital/West China School of Nursing, Sichuan University, Chengdu, 610041 China

**Keywords:** Sleep quality, Effort-reward, Community, Health workers

## Abstract

**Background:**

A chronic state of imbalance between effort and reward can affect sleep quality. However, few studies have explored the relationship between variables in the work-related stress (the effort-reward imbalance model, ERI model) and sleep quality in community health workers in mainland China. We investigated the relationship between ERI and sleep quality in community health workers.

**Methods:**

This cross-sectional study was conducted from September to November 2018 and involved 249 registered doctors and 223 registered nurses. The Pittsburgh Sleep Quality Index (PSQI) was used to evaluate the sleep problems status of the participants. The ERI questionnaire was administered to evaluate job-related stress. Multivariate logistic regression was performed to evaluate the factors related to sleep quality.

**Results:**

The ERI ratio of the 472 health workers was 1.17 ± 0.22, and 273 health workers (57.84%) had PSQI scores > 7.There were statistically significant differences in the effort scores, overcommitment scores and ERI ratio between the health workers with and without sleep problems. The ERI ratio was an independent risk factor for sleep quality; sleep quality, race, type of work, shift work,job title, and personal monthly income were related to the ERI ratio among community health workers.

**Conclusions:**

We found that sleep problems were prevalent, work effort was greater than reward and a positive correlation between effort-reward and sleep quality among community health workers in China. Managers should focus on the factors that influence sleep problems among community health workers, balance the efforts and rewards of work, and reduce the incidence of sleep problems.

## Introduction

Sleep disorders is a common complaint, and the disorder varies across different populations and age groups [1]. The prevalence of insomnia is high worldwide (26.4–39.4% in Asian countries; 10–30% in Western countries) [2–5]. The prevalence of sleep problems is high among health care workers (30–46% in the USA; 37–63.9% in China) [6–9]. Furthermore, many factors, such as sociodemographic and occupational characteristics and occupational risk factors (e.g., shift work and job-related stress), are related to sleep problems [10]. Long-term sleep deprivation may result in serious fatigue, thought retardation, memory loss, slow responses, irritability, depression, suicidal ideation, and occupational accidents [11, 12]. A previous study revealed that insomnia is a significant risk factor for many chronic diseases, such as diabetes, hypertension, cardiovascular disease, and obesity [13]. Many occupational factors may interfere with sleep, sleep problems can, in turn, endanger the health and safety of workers [14]. The literature have demonstrated that experiencing job stress enhances the risk of sleep problems. That means, stress can impair sleep, but a low-quality sleep alters the stress response [15, 16]. The results of the prospective study clearly show a bidirectional relationship between sleep and stress: workers exposed to chronic occupational stress have an increased incidence of sleep problems, and bad sleepers suffer more from occupational stress factors than good sleepers [16]. Therefore, the prevention and control of occupational injuries are challenges for the public health field.

Increasing attention has been given to the development of primary health services in China [17]. Additionally, an imbalance exists in the structural resources. For example, the highest-quality health resources are concentrated in large cities, where Chinese primary health care is still very poor [18]. Most community health workers are busy with basic disease diagnosis and treatment, nursing, chronic disease management, health care for the elderly, maternal health management, child health management, rehabilitation, health education, the management of patients with infectious diseases, and clinical care. These services result in a lack of awareness at the humanistic level and a lack of improvements to medical technology during the service process [19]. However, residents tend to distrust and misunderstand community health care workers, thus affecting the efficiency of work among these professionals, leading to a decline in their sleep quality and a decrease in the safety of their patients [20, 21].

The effort–reward imbalance model proposes similar factors to explain burnout, such as the lack of reciprocity between effort and reward and insufficient levels of respect, esteem, and recognition [22]. The ERI model establishes that workers can feel stress because of an imbalance between perceived effort and reward concerning their work [22]. According to ERI theory, employees expect returns that are aligned with their efforts at work, including income, respect, career development opportunities, and security. The imbalance between effort and reward results in physical and mental impacts, low work efficiency and the dismissal of employees [23, 24]. A chronic state of imbalance between effort and reward can lead to lassitude, anxiety, depression, and other psychological problems that affect sleep quality [24, 25], and also induce metabolic alterations and increase cardiovascular risks [26–28].

Sleep problems have been reported to be prevalent among health workers, and occupational stress can lead to sleep problems [29]. Previous study have suggested that the imbalance between effort and reward may be a direct or an indirect factor leading to poor sleep quality in health workers [30].

In recent years, an increasing number of studies have been conducted on occupational stress and sleep quality. However, to date, few studies have explored the relationship between variables in the ERI model and sleep quality among community health workers in mainland China. Community health workers are more likely to receive lower wages for their efforts [31], which may increase the effort-reward imbalance and decrease sleep quality. The present study investigated the association between sleep quality and ERI among community health workers in mainland China.

## Methods

### Study design

Between September and November 2018, a cross-sectional study using questionnaires was performed among health workers in Chengdu, China, to assess mental health. All the participants were health workers who worked at community hospitals in Chengdu, China. Based on a preinvestigation, 20–30 health workers engaged in clinical activities at each community hospital. Twenty community hospitals in Chengdu City with inpatient departments were randomly selected, and 472 health workers were invited to participate in the survey.

The inclusion criteria were as follows: employed at a community hospital with an inpatient department and inpatient beds of 30–40 in the central urban area of Chengdu, had a signed labor contract with a community hospital; had a doctor’s/nurse’s license; was currently practicing in an inpatient department and providing clinical care; had one or more years of working experience at community hospital [15]. The exclusion criteria were as follows: did not work in inpatient department or provide clinical care; was pregnant or sick or had other personal affairs; had sleep disorder due to alcohol use, disease, or pharmacotherapy; had a family history of sleep disorders; was not willing to participate in the survey.

For data collection, three questionnaires were given to each participant: a questionnaire assessing demographic data, the Effort-Reward Imbalance (ERI) questionnaire, and the Pittsburgh Sleep Quality Index (PSQI) questionnaire. Members of the research team interviewed each participant at their respective work location. The investigator explained the purpose, significance, and relevant instructions for the study and obtained the signed consent form before administering the survey. The questionnaires were provided to the participants electronically through links and quick response codes, and the respondents completed the questionnaires and submitted them online. The data were inputted into EpiData 3.1 software (EpiData – Comprehensive Data Management and Basic Statistical Analysis System; EpiData Association, Odense, Denmark) and then were double-checked.

### Demographic data

This questionnaire was self-designed after a literature review [15]. The participants provided information about their sex, age, race, education background, marital status, chronic illness, type of work, type of contract, work shift, years of work, job title, manager position, personal monthly income and family monthly income.

### Effort-reward imbalance

Studies of occupational stress have considered various theoretical approaches, including the ERI model [23, 24], which emphasizes the nonreciprocal social exchange between costs and gains at work and considers overcommitment, which can cause a state of emotional distress and lead to adverse health outcomes.

The Chinese version of the ERI scale was translated and verified by Li et al. [32]. The Cronbach’s α coefficient of this questionnaire was 0.71, indicating good reliability and validity [32]. The questionnaire assesses external effort (referring to job demand and job duties; 6 items), rewards (referring to money, respect, and job opportunity; 11 items), and overcommitment (referring to the internal pressure of the individual in the process of work; 6 items). These three factors were scored on a scale from 1 to 4 (1 = strongly disagree, 4 = strongly agree). The total score for each component was the sum of the scores for each item; higher scores indicated greater levels of effort/reward/overcommitment [32]. The overall imbalance between effort and reward was obtained using the ERI ratio. The ERI ratio was calculated as [(effort score/reward score) × 0.5454], where 0.5454 is a correction factor. The correction factor is the ratio of the number of items for effort and rewards and adjusts for unequal items on the subscales. The ERI ratio reflects a perception of imbalance between effort and reward. In particular, an ERI ratio > 1 indicated that the individual’s effort outweighed their reward (i.e., it was stressful), an ERI ratio = 1 indicated that the effort was equal to the rewards, and an ERI ratio < 1 indicated that the individual’s reward outweighed their effort.

### Pittsburgh sleep quality index

The Chinese version of the PSQI was used to assess subjective sleep quality. The PSQI was developed by Buysse et al. and Liu et al. [33, 34], who translated the index and verified its reliability and validity. The Cronbach’s α was 0.845 [33]. The PSQI is a scored 18-item self-reported questionnaire that assesses sleep patterns and sleep quality over the previous month. The 18 questions were grouped into seven clinically derived component scores:sleep quality, sleep latency, sleep duration, habitual sleep efficiency, sleep disturbance, sleep medication usage, and daytime dysfunction. Each component was scored on a Likert-type 4-point scale (0 to 3); the components were equally weighted, with higher scores indicating a worse sleep quality. The seven component scores were summed to obtain a global score ranging from 0 to 21. Other researchers have reported that participants with a score of 7 or higher are often considered to have a sleep problem [35]. Thus, in the present study, normal sleep quality was defined as PSQI ≤7, and poor sleep quality was defined as PSQI > 7.

### Statistical analysis

SPSS software (Version 20.0.; IBM Inc., Armonk, NY) was used for the statistical analyses. Quantitative data, such as the ERI score and PSQI score, were presented as arithmetic means and standard deviation, and between-group differences were compared with t-test, analysis of variance, or the nonparametric test. Qualitative data, such as the proportion of participants with a PSQI score > 7, were represented as percentages. Multivariate regression was used to further analyze the relationship between sleep and effort-reward imbalance and confounding factors were considered in the analysis. Multivariate logistic regression was performed to analyze the influencing factors of sleep quality. The dependent variable was the presence or absence of sleep problems, and the independent variables were the ERI ratio and the demographic characteristic variable which *P* > 0.2 was deleted in the model. Multiple linear regression was conducted on the influencing factors of effort-reward imbalance. The ERI ratio was the dependent variable, and the independent variables were with or without sleep problems and the demographic characteristic variable which *P* > 0.2 was deleted in the model. The odds ratios and 95% confidence intervals (CIs) were obtained by logistic regression. A *P*-value < 0.05 was considered statistically significant.

## Results

### Participants

The data from 249 doctors and 223 nurses who were community health workers were analyzed. All the completed questionnaires were valid. The rate of validity of the questionnaires for this study was 100%. The ages of the 472 health workers ranged from 18 to 54 years, with an average age of 36.07 ± 11.31 years. The sample also had the following characteristics: female, 70.8%; age 25 to 44 years, 72.2%; college or above, 92.6%; married, 86.0%; good health, 86.9%; 5–29 years of work experience, 80.5%; and primary/intermediate doctors or nurses, 78.0%.

### Demographic characteristics of the participants, ERI ratios and PSQI scores

The basic characteristics of the participants, ERI ratios and PSQI scores are summarized in Table 1. The overall ERI ratio was 1.17 ± 0.22; 370 (78.39%) health workers had occupational stress (ERI ratio > 1), and the scores for the overcommitment dimension were 17.03 ± 2.28. The total PSQI score was 8.51 ± 3.38, and 273 (57.8%) health workers had a PSQI > 7 (median, 7). Statistically significant differences were found in the ERI ratio among community health workers across the following variables: sex, age, education, marital status, chronic illness, type of work, shift work, years of work experience, job title, manager position and personal/family monthly income (*P* < 0.05). Thus, the following factors led to a greater ERI ratio among community health workers: male participants, 35–44 years of age, bachelor’s degree or above, married, chronic illness, doctor, night shift, 10–19 years of work experience, an intermediate job title, group leader and personal/family monthly income > 10,000 yuan. Statistically significant differences were observed in the PSQI scores among community health workers across the following variables: sex, chronic illnesses, type of contract, shift work, and years of work experience (*P* < 0.05). Thus, male participants, health workers with chronic diseases, those with authorized strength contracts, those working night shifts and those working ≥30 years had greater PSQI scores. No significant differences were observed in other variables (Table 1).

**Table 1:** Demographic characteristics of the participants and total scores for the PSQI and ERI

	*N* (%)	PSQI score	t/F	*P*	ERI ratio	t/F	*P*
Sex			6.233	0.013		9.968	0.002
Male	138 (29.2)	9.11 ± 3.67			1.21 ± 0.24		
Female	334 (70.8)	8.26 ± 3.22			1.15 ± 0.21		
Age range, y			2.476	0.061		11.458	0.000
18–24	42 (8.9)	7.21 ± 2.91			1.03 ± 0.18		
25–34	164 (34.7)	8.48 ± 3.38			1.14 ± 0.20		
35–44	177 (37.5)	8.70 ± 3.32			1.23 ± 0.23		
45–54	89 (18.9)	8.79 ± 3.59			1.16 ± 0.20		
Race			0.334	0.564		3.621	0.058
Ethnic Han	453 (96.00)	8.49 ± 3.40			1.17 ± 0.22		
Others	19 (4.00)	8.94 ± 2.71			1.07 ± 0.28		
Education			1.380	0.253		11.489	0.000
Polytechnic school	35 (7.4)	8.14 ± 3.61			1.09 ± 0.13		
College	228 (48.3)	8.30 ± 3.33			1.13 ± 0.21		
Bachelor’s degree or above	209 (44.3)	8.79 ± 3.37			1.22 ± 0.23		
Marital status			3.907	0.452		3.087	0.002
Married	406 (86.0)	8.55 ± 3.46			1.18 ± 0.22		
Single	66 (24.0)	8.26 ± 2.81			1.10 ± 0.17		
Chronic illness			7.748	0.006		5.991	0.015
Yes	62 (13.1)	9.61 ± 3.51			1.23 ± 0.19		
No	410 (86.9)	8.34 ± 3.33			1.16 ± 0.22		
Type of work			3.717	0.054		28.627	0.000
Doctor	249 (52.8)	8.79 ± 3.48			1.22 ± 0.24		
Nurse	223 (47.2)	8.19 ± 3.24			1.11 ± 0.18		
Type of contract			3.937	0.020		2.766	0.064
Long-term contract	186 (39.4)	8.21 ± 3.21			1.14 ± 0.21		
Permanent contract	39 (8.3)	7.54 ± 3.02			1.13 ± 0.19		
Authorized strength	247 (52.3)	8.89 ± 3.51			1.19 ± 0.23		
Shift work			9.862	0.002		29.256	0.000
Day shift	327 (69.3)	8.19 ± 3.33			1.13 ± 0.21		
Night shift	145 (30.7)	9.23 ± 3.39			1.25 ± 0.22		
Years of work experience			4.810	0.001		8.341	0.000
< 5	65 (13.8)	6.92 ± 2.86			1.03 ± 0.20		
5–9	127 (26.9)	8.90 ± 3.52			1.18 ± 0.20		
10–19	150 (31.8)	8.81 ± 3.10			1.21 ± 0.22		
20–29	103 (21.8)	8.40 ± 3.55			1.18 ± 0.23		
≥ 30	27 (5.7)	9.26 ± 3.69			1.14 ± 0.15		
Job title			2.268	0.080		12.587	0.000
Registered	39 (8.3)	7.49 ± 3.25			1.05 ± 0.18		
Primary	225 (47.7)	8.34 ± 3.36			1.13 ± 0.21		
Intermediate	143 (30.3)	8.86 ± 3.39			1.23 ± 0.22		
Subsenior	65 (13.8)	8.94 ± 3.37			1.22 ± 0.24		
Manager position			2.164	0.116		6.483	0.002
No	314 (66.5)	8.28 ± 3.34			1.14 ± 0.21		
Group leader	38 (8.1)	8.89 ± 3.62			1.23 ± 0.22		
Director/Head doctor, nurse	120 (25.4)	8.98 ± 3.36			1.21 ± 0.23		
Personal monthly income, yuan			1.542	0.189		7.073	0.000
≤ 3000	59 (12.5)	7.73 ± 3.86			1.07 ± 0.22		
3001-5000	197 (41.7)	8.64 ± 3.21			1.14 ± 0.21		
5001-8000	165 (35.0)	8.80 ± 3.42			1.21 ± 0.22		
8001-10,000	36 (7.6)	7.94 ± 3.20			1.21 ± 0.19		
> 10,000	15 (3.2)	7.93 ± 3.17			1.29 ± 0.17		
Family monthly income, yuan			0.335	0.854		2.688	0.031
≤ 3000	52 (11.0)	8.60 ± 3.67			1.13 ± 0.22		
3001-5000	129 (27.3)	8.77 ± 3.36			1.18 ± 0.22		
5001-8000	112 (23.7)	8.46 ± 3.13			1.16 ± 0.23		
8001-10,000	90 (19.1)	8.32 ± 3.40			1.12 ± 0.18		
> 10,000	89 (18.9)	8.33 ± 3.53			1.21 ± 0.22		

### Comparison between doctors and nurses with different demographic characteristics in ERI and PSQI measures

There were statistically significant differences between the doctors and nurses in the effort score, reward score, overcommitment score and ERI ratio (Table 2). Additionally, nurses perceived more rewards, less effort and less overcommitment than doctors (*P <* 0.01). Doctors had a higher ERI ratio than nurses (*P* = 0.001). The PSQI scores were similar between the doctors and nurses.

**Table 2:** Comparison of the ERI and PSQI measures between doctors and nurses

Characteristics	Doctors	Nurses	*t*	*P*
	M ± SD	M ± SD		
Effort (scores)	19.31 ± 3.34	18.19 ± 3.03	14.427	0.000
Reward (scores)	29.37 ± 2.93	30.09 ± 2.39	8.451	0.004
Overcommitment (scores)	17.28 ± 2.30	16.75 ± 2.23	6.472	0.011
ERI ratio	1.22 ± 0.24	1.11 ± 0.18	28.627	0.000
PSQI scores	8.79 ± 3.48	8.19 ± 3.24	3.717	0.054

### Associations between ERI scale scores and sleep disturbances

Across the four factors of the ERI, we found that the effort score, overcommitment score, and total ERI ratio were significantly different (*P =* 0.000, Table 3) between the health workers with poor sleep quality (PSQI > 7) and those with normal sleep quality (PSQI ≤7).

**Table 3:** ERI factors and sleep disturbances

	Nonsufferers ^A^ (Mean ± SD)	Sufferers ^B^ (Mean ± SD)	*t*	*P*
Effort (scores)	17.32 ± 3.00	19.84 ± 3.00	−9.013	0.000
Reward (scores)	29.46 ± 2.49	29.89 ± 2.84	−1.741	0.082
Overcommitment (scores)	15.88 ± 2.06	17.86 ± 2.07	−10.292	0.000
ERI ratio	1.08 ± 0.20	1.22 ± 0.21	−7.496	0.000

^A^ Nonsufferers indicate those with PSQI scores ≤7; ^B^ sufferers indicate those with PSQI scores > 7

### Logistic regression analysis of multiple factors related to sleep quality

Logistic regression analysis showed that the ERI ratio, type of contract, shift work were the main risk factors for sleep problems in community health workers. Logistic regression analysis was also performed, stratified by type of health worker (doctor and nurse), the ERI ratio was the main risk factor for sleep problems in community doctor or nurse (Table 4).

**Table 4:** Logistic regression analysis of factors related to sleep quality

Model	Independent variable	β	Wald	*P*	OR (95% CI)
Model 1^A^	ERI ratio	−1.289	6.546	0.011	0.275 (0.103, 0.740)
	Type of contract		6.375	0.041	
	Permanent contract	0.887	6.133	0.013	2.249 (1.203, 4.903)
	Authorized strength	0.666	3.346	0.067	1.946 (0.954, 3.970)
	Shift work	0.482	5.162	0.023	1.619 (1.068, 2.454)
	Constant	1.478	1.091	0.175	
Model 2^B^	ERI ratio	−1.025	3.916	0.048	0.359 (0.130, 0.990)
	Constant	2.412	5.710	0.017	
Model 3^C^	ERI ratio	0.997	9.941	0.002	2.711 (1.458,5.308)
	Constant	−1.654	7.546	0.006	

^A^ Logistic regression analysis of factors related to the sleep quality of health workers. ^B^ Logistic regression analysis of factors related to the sleep quality of doctors. ^C^ Logistic regression analysis of factors related to the sleep quality of nurses

### Linear regression analysis of multiple factors related to the ERI ratio

The results of linear regression analysis showed that sleep quality, race, type of work, shift work, job title, and personal monthly income were the main factors that affected the ERI ratio among community health workers. Linear regression analysis was also performed, stratified by type of health worker (doctor and nurse), sleep quality, shift work and job title were the main factors that affected the ERI ratio for community doctors; sleep quality, shift work and personal monthly income were the main factors for community nurses (Table 5).

**Table 5:** Linear regression analysis of factors related to the ERI ratio

Model	Independent variable	Unstandardized Coefficients		standardized regression coefficient	t	*P*
		β	standard error			
Model 1^A^	Constant	0.902	0.075		12.073	0.000
	Sleep quality	0.122	0.018	0.274	6.727	0.000
	Race	−0.108	0.045	−0.097	−2.405	0.017
	Type of work	−0.079	0.018	−0.180	−4.419	0.000
	Shift work	0.097	0.019	0.205	5.013	0.000
	Job title	0.045	0.017	0.128	2.734	0.007
	Personal monthly income	0.025	0.011	0.104	2.303	0.022
Model 2^B^	Constant	0.622	0.069		9.407	0.000
	Sleep quality	0.150	0.027	0.306	5.467	0.000
	Shift work	0.103	0.028	0.202	3.625	0.000
	Job title	0.083	0.016	0.285	5.105	0.000
Model 3^C^	Constant	0.746	0.053		14.047	0.000
	Sleep quality	0.083	0.022	0.232	3.730	0.000
	Shift work	0.094	0.025	0.236	3.798	0.000
	Personal monthly income	0.051	0.013	0.236	3.904	0.000

^A^ Linear regression analysis of factors related to the ERI ratio of health workers. ^B^ Linear regression analysis of factors related to the ERI ratio of doctors. ^C^ Linear regression analysis of factors related to the ERI ratio of nurses

## Discussion

The present study showed that sleep problems were prevalent among community health workers in China. Additionally, we found a higher risk of sleep problem among male participants, those with chronic illness, those with authorized strength contracts, those who worked night shifts, and those who had worked ≥30 years. More importantly, the study indicated that, as the health workers’ ERI ratios increased, sleep quality progressively worsened (Table 1), indicating that job stress affects the sleep quality of health workers [6, 8, 35].

Our research showed that community doctors perceived higher levels of effort and overcommitment and lower levels of rewards than community nurses. This finding is inconsistent with other study findings [36, 37]. The reward score comprises three components: financial and career-related aspects, esteem-related rewards, and the gratification of job security [24]. As the primary providers of health services, doctors have a heavy burden. Some general practitioners (GPs) came from general hospitals, and they were exposed to several factors that are inferior to those of general hospitals, such as work overload, time pressures, role conflicts, lower compensation and fewer career development opportunities. Promotions and other normal conditions also limit GP development and effort-reward imbalances; thus, they encounter more job stress than nurses, and their job satisfaction is not high. In contrast, the jobs of nurses are relatively clear in the community; thus, their job satisfaction is higher than that of GPs [31, 38]. Regardless of the unsafe clinical environment, health workers are always committed to providing timely health services without hesitation or reservations in China, contributing to the imbalance between effort and reward and leading to higher job stress [39, 40]. Our results showed that the ERI ratio of community health workers was 1.17 ± 0.22, indicating that community health workers generally perceived more effort than rewards. We found a higher ERI ratio among those with the following demographic characteristics: males, 35–44 years, bachelor’s degree or above, married, chronic illness, doctors, worked night shifts, working ≥30 years, intermediate job title, group leader, and monthly income > 10,000 yuan. This finding is consistent with other study findings [41, 42]; the occupational task and stress response for the male participants were higher than those for the female participants and higher in those with a high education level than in those with a low education level. The scores on the overcommitment dimension were 17.03 ± 2.28, which was higher than that reported in previous studies [42, 43]. Inadequate compensation is a concept incorporated into the ERI model: high effort and low compensation lead to high stress [35]. In the present study, 370 (78.39%) health workers had a high ERI (ERI ratio > 1), which was associated with sleep problem,consistent with previous study [44]. Another research has suggested that a high ERI ratio (versus low ERI) is associated with poorer psychosocial care and even more errors [45]. Moreover, many previous studies have shown that effort–reward imbalance can lead to adverse health outcomes [33, 46].

With health system reforms, the connotation and work volume of community health workers have continuously increased, and occupational stress will inevitably increase. The participants in the present study had higher levels of occupational stress, likely because the study was conducted in southwestern China, which is less economically developed; thus, the rewards for community health workers are far lower than those in coastal areas. Additionally, doctor-patient relationships are becoming increasingly fragile in China [47]. Using doctor-patient relationships to evaluate the costs and rewards of a doctor’s professional and emotional investment is unfair because these fragile relationships result in resource depletion, which is closely related to job stress, burnout and depression in health workers [48]. Therefore, managers should adopt measures to reduce the incidence of occupational stress among health workers at community hospitals.

Health workers with poor sleep quality had higher levels of perceived effort, higher levels of perceived overcommitment, and higher ERI ratios than those with good sleep quality (Table 3). This finding is consistent with existing study findings [49]. Table 4 shows that the ERI ratio is the main risk factor for sleep disturbances in community health workers, and the same result was found for doctors and nurses. Overall, 78.39% of health workers had occupational stress (ERI ratio > 1), higher than that reported by Ge et al. (64.7%) [50]. This result can be explained by China’s national conditions and work characteristics of the community health sector. In China, the need for health services is increasing with the development of the economy, particularly at community hospitals [15], and the nature of psychosocial work environments varies considerably between hospitals and primary care [51]. Community health workers are generally considered less competent than those at general hospitals, not only doctors but also nurses; this situation requires community health workers to expend more effort to earn respect from ordinary people and achieve overall organizational goals. The study showed that overcommitment among community health workers was high, indicating that they might overestimate their capabilities and play more effort into finishing work that is beyond their abilities [50].

Further analysis indicated that sleep quality, race, type of work, shift work, job title and personal monthly income were the main risk factors for a higher ERI ratio in community health workers, but a difference was observed between doctors and nurses (Table 5). Sleep quality and shift work were the same risk factors for a higher ERI ratio for doctors and nurses. The difference is likely due to the work characteristics of community doctors and nurses. Night nurses are under more job stress than night doctors, but nurses are paid far less than doctors. A previous study recognized that shift work could affect not only nurses’ personal health but also the quality of their work and their patients’ psychological health and treatment, even result in mistakes and accidents [52]. Previous studies showed that reward (rather than effort) is a strong determinant of employees’ job satisfaction and quality of care [53, 54]. Community doctors are under a high-stress occupation, lack promotion and learning opportunities, decreasing their job satisfaction. Another study showed that high levels of satisfaction with the nature of one’s work positively affected physical and mental health [55]. If the efforts and rewards at work cannot be balanced, professionals will find their jobs difficult, causing occupational stress and affecting sleep quality. Overcommitment and effort–reward imbalance have been identified as important occupational stress factors that negatively contribute to the psychological and physical health and well-being of employees [56].

This study confirmed the hypothesized bidirectional forward associations between sleep and stress: community health workers exposed to chronic job stress have an increased incidence of sleep problems. This results are similar observations from the Swedish Longitudinal Occupational Survey of Health [57]. In fact, being a cross-sectional study, no one can tell whether workers had job stress first, or sleep problems first. Our sample is slightly less homogeneous, although the work performed by community health workers are basically the same, they still have different tasks, some with night work, some without, some managerial, some not. The study results may not be possible to generalize the findings to other cultures and geographic regions in China.

Community health workers report higher levels of ERI, resulting in occupational stress, which leads to sleep problems and diseases. Therefore, organizational interventions, such as increasing rewards and improving doctor-patient relationships, should be considered in China.

This study had several limitations that may have affected the outcomes. The main limitation of our study is convenience sample, but not having a randomized sampling method resulting a small sample size if we consider that we are referring to Chinese community workers, at the same time the lack of a control group limits the generalization of the results. Another limitation is assessing community health workers for serious sleep problems and comparing the results across individuals with different professional functions should be performed.

## Conclusions

We found that sleep problems were prevalent among health workers at community hospitals in Southwest China. The main risk factors for sleep disturbances in health workers was ERI ratio. The ERI affects sleep quality in Chinese community health workers, such that higher ERI ratios lead to a worse sleep quality. Sleep disturbances may lead to a lower quality of life and lower work efficiency levels for health workers, increasing the potential for errors or medical malpractice. Therefore, awareness and interventions are required to reduce job stress at community hospitals. Additional research on this topic is also required.

## Data Availability

The datasets used and analysed during the current study are available from the corresponding author on reasonable request.

## Abbreviations

PSQI: Pittsburgh Sleep Quality Index
ERI: Effort-Reward Imbalance
